# Disease-Related Detection with Electrochemical Biosensors: A Review

**DOI:** 10.3390/s17102375

**Published:** 2017-10-17

**Authors:** Ying Huang, Jin Xu, Junjie Liu, Xiangyang Wang, Bin Chen

**Affiliations:** 1 Chongqing Key Laboratory of Non-linear Circuit and Intelligent Information Processing, College of Electronic and Information Engineering, Southwest University, Chongqing 400715, China; hanhanblala@email.swu.edu.cn (Y.H.); wx42633@email.swu.edu.cn (J.X.); bd630985@email.swu.edu.cn (J.L.); 2 Key Laboratory of Luminescent and Real-Time Analytical Chemistry (Southwest University) Ministry of Education, College of Pharmaceutical Sciences, Southwest University, Chongqing 400715, China; 3 CET-College of Engineering and Technology, Southwest University, Chongqing 400715, China; wang19960207@email.swu.edu.cn

**Keywords:** electrochemical biosensor, disease detection, biomolecules

## Abstract

Rapid diagnosis of diseases at their initial stage is critical for effective clinical outcomes and promotes general public health. Classical in vitro diagnostics require centralized laboratories, tedious work and large, expensive devices. In recent years, numerous electrochemical biosensors have been developed and proposed for detection of various diseases based on specific biomarkers taking advantage of their features, including sensitivity, selectivity, low cost and rapid response. This article reviews research trends in disease-related detection with electrochemical biosensors. Focus has been placed on the immobilization mechanism of electrochemical biosensors, and the techniques and materials used for the fabrication of biosensors are introduced in details. Various biomolecules used for different diseases have been listed. Besides, the advances and challenges of using electrochemical biosensors for disease-related applications are discussed.

## 1. Background

**Figure 1:**
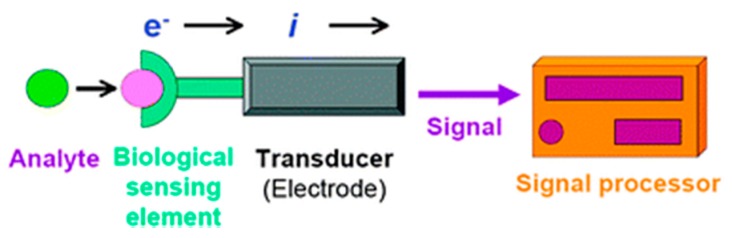
Scheme of a biosensor with electrochemical transducer [2]. Copyright 2010. Reproduced with permission from The Royal Society of Chemistry.

Information from the outer world is obtained in virtue of the sense organs. A sensor is a device used to gather information registered by some biological, physical or chemical change, and then convert the information into a measurable signal. Typically, it contains a recognition element and a transducer. Electrochemical biosensors, as a subclass of biological sensors, consist of a biological sensing element and an electrochemical transducer (see Figure 1). The recognition element (enzymes, antibodies, DNA/RNA, tissues or other biomolecules) reacts selectively with the target analyte, and as a result, an electrical signal is produced and then transmitted via the transducer to the signal processor. After a series of steps, such as amplification and separation, useful information becomes available. Electrochemical biosensors are widely developed, and some of them have reached the commercial stage and are routinely used in environmental and agricultural applications, and especially, in clinical laboratory and industrial analysis [1]. The electrochemical glucose biosensor is a typical prototype widely utilized to monitor the blood glucose concentrations of diabetes patients and for the detection of celiac disease [2], breast cancer [3,4], prostate cancer [5], hepatitis B virus [6], etc. Electrochemical biosensors can be roughly divided into two categories based on the nature of the biological recognition process: biocatalytic devices and affinity sensors [7]. Biocatalytic sensors incorporate enzymes, cells, tissue slices as the sensing elements that recognize the target analyte and selectively increase the reaction rate significantly relative to an uncatalyzed reaction [8]. After that, some electroactive species or some other detectable outcome will be produced. In cases that the enzyme of the substation is not available, too expensive or the analyte is not available in living systems, an affinity sensor is another reasonable alternative. This biosensor is based on the interaction between the target analyte and biochemical elements such as antibodies, nucleic acids (DNA/MicroRNA), etc. Immunosensors and electrochemical DNA hybridization biosensors are two typical examples of affinity sensors [1].

Electrochemistry is a surface technique that can offer certain advantages, and it does not depend on the reaction volume. Generally, electrochemical biosensors offer advantages such as high specificity of their biological recognition process, low background noise and better signal to noise ratios. Moreover, with this method measurements need very small sample volumes. Electrochemical detection is widely chosen for the transduction of biosensors due to its low cost, simplicity of construction, user-friendliness and portability [9]. Recently, the use of voltammetry/amperometry techniques is becoming more common in biosensors. These techniques apply a potential to a working electrode versus a reference one, and measure the current by means of an electrochemical reduction or oxidation at the working electrode. Therefore, compared to traditional assay methods that require highly trained operators or sophisticated instrumentation, the development of sensitive immunoassays or sandwich assays for biomarker detection has great clinical significance. This review focuses on the developing methods for electrode fabrication, immobilization techniques, measurement tools and relative materials associated with electrochemical biosensors that are used as analytical tools for disease-related detection. The detection of biomarkers of diseases in their early stage is of vital importance. The review also discusses some limitations, and describes the recent work on electrochemical biosensors.

## 2. Electrochemical Biosensors

### 2.1. Different Recognition Elements

**Figure 2:**
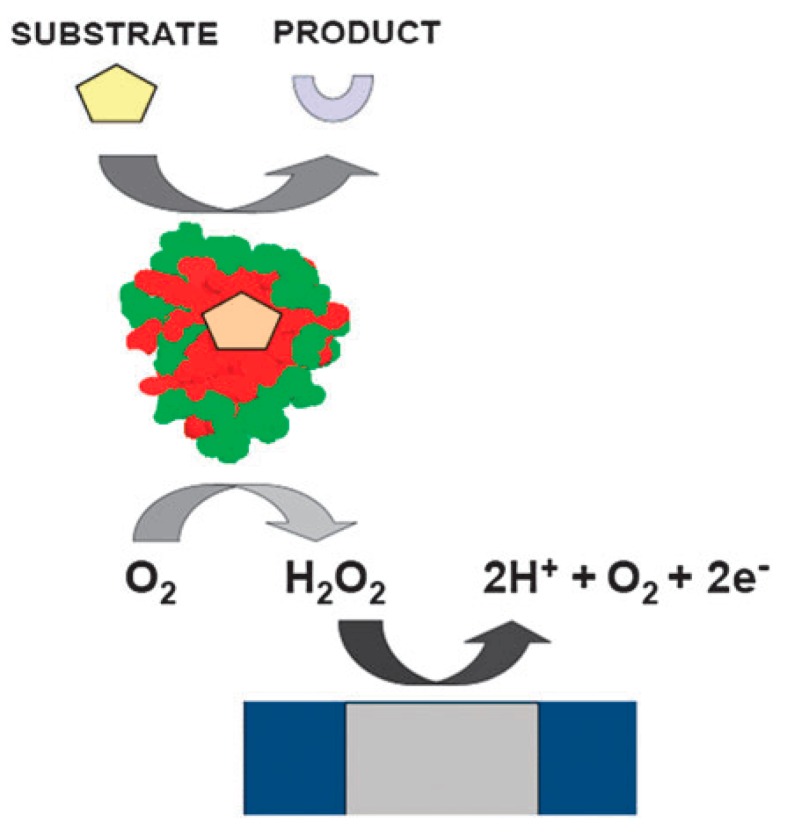
First-generation biosensor that depends ambient oxygen with amperometric detection [2] Copyright 2010. Reproduced with permission from The Royal Society of Chemistry.

Generally, electrochemical sensors incorporate a biological recognition element and an electrochemical transducer. Based on the nature of the biological recognition process, there are two main categories: biocatalytic devices and affinity sensors [1]. Biocatalytic sensors can be divided into several types according to the material of their recognition elements, including enzymes, whole cells [10,11], tissues slices, etc. Different recognition components selectively recognize the target analyte, catalyze the reaction of the substrate and produce some electroactive species. Among these, enzyme-based biosensors are the most common category, and three generations of these have evolved. The first-generation sensors (see Figure 2) need the ample and constant presence of ambient oxygen as a co-substrate, which means the oxidizing agent is determinant and as a result, some problems occur. In the first place, oxygen is not very soluble in aqueous solution, leading to a limitation of the rate of the reaction and production of electroactive species. Moreover, the use of oxygen as the oxidizing agent limits the variety of substrates that can be analyzed because some target analytes can’t react with oxygen. Further, since the enzyme active sites are usually buried and not easily accesible to the analyte, the electrons produced in the enzyme-catalyzed reaction can’t always be rapidly and efficiently transferred to the electrode surface, thereby influencing the communication between the enzyme and the transducer. The famous Marcus theory of electron transfer indicates that electron transfer decays exponentially with distance.

The second-generation, as an improved one, incorporates a manually added component called mediator, which is nontoxic and unreactive with oxygen, as an alternative choice to oxygen. Biosensors with a mediator perform much better relative to the first-generation ones. This technique avoids the influence of the low solubility of oxygen in aqueous solution as well as the dependence on oxygen. Meanwhile, it is possible to choose a mediator with more suitable oxidation potential among a variety of oxidizing agents. Second-generation biosensors have advantages including high specificity.

**Figure 3:**
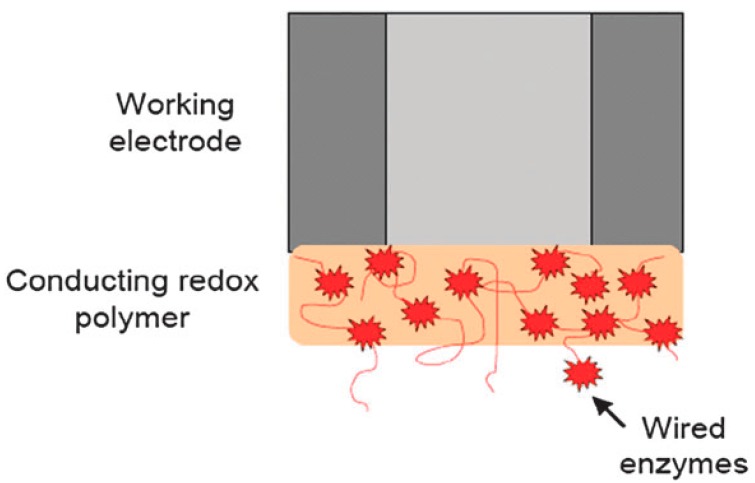
Schematic diagram of third-generation catalytic biosensor [2]. Copyright 2010. Reproduced with permission from The Royal Society of Chemistry.

More recently, third-generation biosensors (see Figure 3) with a lot of improvements have come out, and have been widely applied. The main difference of the third-generation ones relative to the previous sensors is that the enzyme is immobilized on the electrode surface as well as the mediator, instead of manually adding it into the aqueous solution. The enzyme and mediator were immobilized on a conducting [12] or a redox polymer, preventing the enzymes and the mediator from diffusing everywhere. The third-generation sensors perform much better in transporting electrons between the active site of the enzyme and the electrode surface, and the efficiency is improved. Moreover, this self-contained nature reduces the time-consuming and high cost requirements.

As some isolated enzymes are expensive and not easy to obtain, biosensors based on tissues and bacteria may be a suitable alternative. These biosensors incorporate plant tissue and bacteria that act as the recognition components. The enzymes exist in the tissue of bacteria, and the electrode works in a similar way as an enzyme electrode. It performs well due to the high catalytic activity of its enzymes relative to the isolated enzymes that must operate outside their natural living conditions, and it is characterized by high stability. However, on the contrary it requires a long test time.

In addition to the above, for disease-related detection, affinity biosensors based on antibodies or nucleic acids are another approach. Immunosensors are a typical kind which are based on immunoassays. An immunosensor incorporates an antibody as the electrochemical recognition element and a transducer based on a light-sensitive material, which may include several categories such as fiber optical sensors [13], surface plasmon resonance (SPR) sensors [14] and so on. Based on the highly selective recognition of antibodies for antigens, immunosensors have been manufactured and are widely applied in monitoring diseases related to proteins, as well as in environmental [15], agricultural [16], processed food and beverage industry applications [17]. A wide range of interfacial strategies have been developed and applied for the selective detection of biomarkers. Once some foreign molecules like proteins that have high molecular weight (antigens) or chemical compounds invade an organism, antibodies are able to recognize and selectively bind with them, and ultimately get rid of them. The binding process between antibodies (AB) and antigens (AG) is called immunoassay, which has high specificity and sensitivity. Recently, the use of immunosensors to detect biological agents and harmful chemicals in defense applications has gradually become common [18]. The DNA hybridization biosensor is another typical category of affinity biosensors which incorporates single-stranded, 15–40 bases long DNA or RNA oligonucleotide sequences as the recognition components, called aptamers. Aptamers selectively bind with the target analyte such as small sections of macromolecules. DNA hybridization biosensors are widely used for diagnosis of genetic or infectious diseases all over the world [19]. When preparing a DNA biosensor, the aptamer production, which requires single-stranded and 15–40 bases long DNA or RNA oligonucleotide sequences, is of vital importance. The aptamer production is based on systematic evolution of ligands by exponential enrichment (SELEX) and polymerase chain reaction (PCR). In the first place, a large quantity of random DNA or RNA sequences are screened in a SELEX process and the sequences that are capable to selectively binding with the low molecular weight organic, inorganic or protein targets stand out. As a result of this binding event, the aptamer is folded into a complicated three-dimensional shape that allows the aptamer molecule to bind tightly and selectively to the target molecule. After the first round of SELEX, the unbound DNA or RNA sequences are removed and the bound nucleic acid strands are replicated for another round of SELEX, with the help of PCR. After several cycles of SELEX, the chosen aptamers may be modified to increase the affinity to the target analyte, as well as stability to pH and temperature. As the sequences of aptamers are known, they can be produced in large quantities [20]. The aptamer binds with the target DNA in a process called hybridization, and an electrical signal is generated. When an electrochemical indicator binds preferentially to the DNA duplexes, an electrochemical signal forms and shows the hybridization. There are two ways to monitor the hybridization, including label electrochemical measurements and label-free ones. Sometimes, colloidal gold that belongs to the category of nanoparticle labels is a good choice for quantifying the binding between the aptamer and the target DNA. Electrochemical measurement of catalytic products from a capture enzyme label such as alkaline phosphatase can also be utilized for the measurement of hybridization. Label-free electrochemical measurement induce some changes in conductivity at the electrode surface, and is also capable of monitoring the hybridization. In addition, aptamers require small variations of the surrounding environment such as pH and temperature, and sensor surface coverage by the DNA probe is also of vital importance in minimizing nonspecific binding. In order to retain the stability, and reactivity of the aptamer to the target DNA, condition such as pH, temperature, and ionic strength have to be strictly controlled.

### 2.2. Immobilization Strategies

In the design of biosensors, the immobilization of biomolecules on the transducer surface is an important procedure. Different immobilization techniques may contribute to differing biosensor sensitivity and stability. Various immobilization methods have been used to develop optical, electrochemical or gravimetric enzymatic biosensors [20]. The choice of immobilization method depends on the recognition sensing element and the surface. Electrochemical biosensors require simplicity [21] and reproducibility [22], while avoiding non-specific binding as much as possible. Various classical techniques reported in the literature include adsorption [23], covalence [24], entrapment [25], cross-linking [12] and affinity [26]. Generally, adsorption is considered the simplest and easiest immobilization method. It was based on weak bonds such as Van der Waal’s forces and electrostatic and/or hydrophobic interactions. Immobilization by adsorption is a dynamic process with continuous adsorption and desorption [27] from the surface. It is reversible, and does not destroy the molecular structure or the activity of the enzyme. No functionalization of the support is involved. In the process, taking an enzyme as an example, the enzyme is dissolved in solution and the solid support is put in contact with the solution for a set period of time, then, specific washing buffers clean the remaining or excess unabsorbed enzyme. However, this immobilization suffers from biomolecule desorption due to the weak-binding. External factors, such as temperature, ionic strength and pH [28], may lead to desorption too. Also, adsorption leads to deactivation of the biomolecules and may result in an activity loss. Dixit et al. [29] studied non-specific protein loss due to adsorption on sample tube surfaces and altered immunogenicity. By contrast, covalent immobilization is relatively strong. For antibody immobilization, for instance, it is performed with surfaces that possess pendant reactive chemical functionalities, since biomolecules, such as proteins and nucleic acids, possess functional groups that are easily manipulated to capture these biomolecules, Dixit et al. [30] studied the antibody biomolecule immobilization from the perspective of the functional groups of an antibody, such as carboxyl, amines, sulfhydryl, etc. The authors have demonstrated the important prerequisites for designing an efficient immobilization strategy, including: (a) the antibody must retain its functional conformation and activity after immobilization; (b) the surface used for attachment should either possess or be amenable to the grafting of desired chemical functionalities, and (c) attachment must occur easily with maximum efficiency and minimum loss in antibody-binding capacity. In addition, Shen et al. [31] discussed the effect of antibody immobilization on the parameters of the performance of an immunoassay. Conventional site-directed antibody immobilization techniques have been also studied [32], and their advantages and disadvantages are listed directly. In addition to the above, for immobilization of probe DNA onto an electrode surface, a solution based on the covalent method which immobilizes target DNA on Au electrodes which have been functionalized by direct coupling of sol-gel and self-assembled technologies was used [33,34]. Polymers are becoming inseparable from biomolecule immobilization strategies and biosensor platforms [35,36]. In the fabrication of some biosensors, enzymes are immobilized by entrapment in three-dimensional matrices, which could be an amphiphilic network composed of polydimethylsiloxane (PDMS), an electropolymerized film (electrochemical polymerization), a photopolymer (photopolymerization), a carbon paste or a polysaccharide. Ionescu et al. used membranes of polymers as well as sol-gel entrapment. Glutaraldehyde and carbodiimide are the most exploited techniques for covalent attachment. Cross-linking is another well-known approach used in glucose biosensors [12], in which enzymes are immobilized with glutaraldehyde or other bifunctional agents (e.g., glyoxal, hexamethylenediamine). Affinity bonds between an activated support and specific groups of the protein sequence are created. Since this method allows controlling biomolecule orientation, it avoids enzyme deactivation and active site blocking. More recently, other immobilization strategies aiming to lower the detection concentration or improve the system performance of biosensors are reported. These techniques are demonstrated to be easy to implement, making the surface optimiztion possible.

### 2.3. Electrical Communication between the Biomolecules and the Transducer

**Figure 4:**
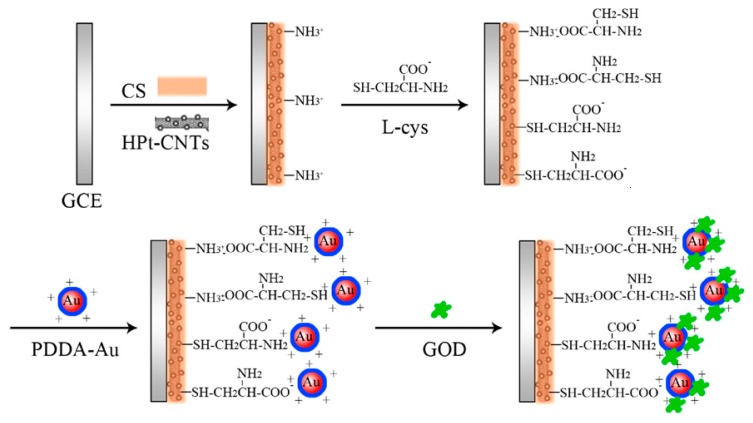
Schematic of the fabrication process of the biosensor on which the multiwall CNTs (HPt-CNTs) has been decorated with unique hollow nanostructured Pt [37]. Copyright 2011. Reproduced with permission from Elsevier B.V.

One problem of electrochemical biosensors is how to establish the electrical communication between the recognition element and the electrode surface [37]. Broadly speaking, there are two approaches for this. One is based on electrochemical mediators such as natural enzyme substrates (e.g., oxygen) and artificial redox mediators (e.g., conductive polymers). This approach was widely used in first- and second-generation biosensors. Third-generation biosensors are based on another approach, which is a direct way related to electron transfer of proteins, serving as a transduction element. One field that offers great potential for electron transfer applications is that comprising redox enzymes or proteins [38]. Protein-film voltammetry (PFV) is a common technique for achieving the direct electron transfer (DET) used during the early years [39]. PFV scans a film of proteins absorbed on the electrode surface. The protein is immobilized on the electrode surface, then, signals are obtained from small sample quantities. PFV has been applied in the field of protein chemistry, leading to many discoveries. It has advantages over conventional voltammetry in which the protein molecules are free in solution, including the fine-redox status of the entire sample, waveform definition, sensitivity and fast reactions. Later, nanomaterials have been extensively used in biosensors to realize direct electron transfer. Decorating carbon nanotubes (CNTs) with metallic nanoparticles is of great potential for direct electron transfer [40,41,42]. CNT-assisted biosensing technologies, such as DNA-hybridization, protein-binding, antibody-antigen and aptamers have been reviewed. It is demonstrated that CNTs have the potential to revolutionize numerous applications where nanosized metallic and/or semiconducting components are required [43]. For example, glucose biosensors combined the CNTs have been decorated with Au-coated Pd nanotubes [44], gold nanoparticles [45] and Pt nanomaterials [46]. Particularly, Pt nanomaterials with hollow interiors are promising owing to their capabilities to enhance electron transport and increase the surface area. Extending the surface area of the cathode powder is an effective approach to increasing the activity of an electrode [47]. A biosensor on which the multiwall CNTs (HPt-CNTs) decorated with unique hollow nanostructured Pt has led to the achievement of direct electron transfer (see Figure 4) [37].

Direct electron transfer leads to a closer integration between the recognition element and the transducer of an electrochemical biosensor and thus increases the sensitivity of electrochemical biosensors. However, there are still some limitations to overcome, such as the number of proteins (or enzymes), that indicate the phenomenon of electron transfer on the signal transducer, and the kinetics of electron transfer between the recognition element and electrode surface also limit the use of these biosensors.

### 2.4. Materials Used in Electrochemical Biosensors

**Figure 5:**
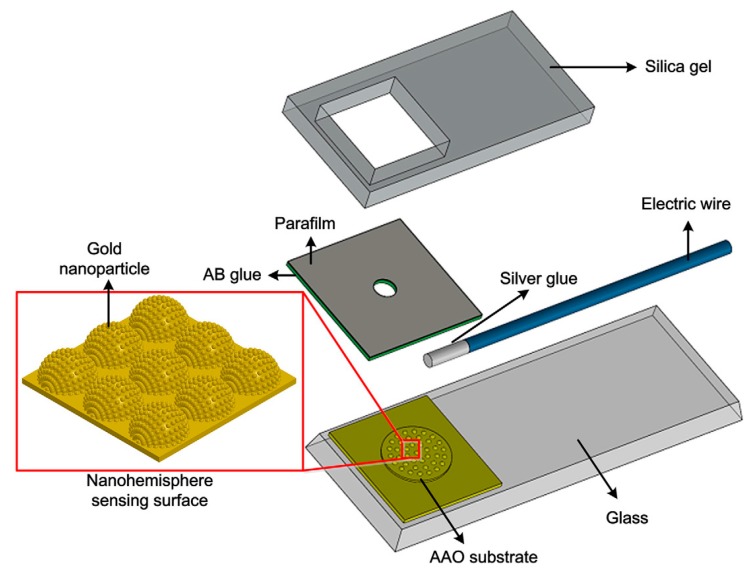
Schematic of the fabrication process of nanostructured biosensor [52]. Copyright 2014. Reproduce with permission from Elsevier Inc.

Various materials including semiconductors, ceramics, metals, organic materials and metamaterials have been widely used in the manufacture of various types of sensors. In recent years, the development of nanomaterials has rapidly attracted attention [46,48]. A large quantity of nanomaterials inclusing nanoparticles [49], nanowires and nanotubes in different sizes and shapes have been proposed for this purpose. These types of nanomaterials are accessible [50] and gradually became popular for various aspects of the electrochemical biosensor detection system, including in capture probes, reporting molecules and electrode fabrication and coatings. Rusling et al. [51] described the history of the use of nanoparticles in electrochemical protein sensors, and discussed fabrication of nanostructured sensors and arrays. Figure 5 shows the fabrication scheme of a nanostructured biosensor.

Besides, metal semiconductor nanoparticles have developed rapidly accompanied by nanomaterials [53,54]. Typical metal nanoparticles such as colloidal gold and inorganic nanocrystals have been widely utilized in biological tagging schemes. Metal nanoparticles also facilitate the contact of enzymes’ active sites and the electrode surface. Gold nanoparticles show excellent properties for optimizing the performance of biosensors by acting as electron relays [55]. Some immobilization strategies have pursued metal nanomaterials, and incorporate them onto the electrode surface by techniques such as adsorption, entrapment into polymers and self-assembly. Printed gold electrodes can be generated by ink-jet printing of gold nanoparticle ink followed by sintering. A continuous functional gold electrode is achieved in this process, but it has a high tendency to dissolve away at potentials above 0.9 V. Also, the surface area characterization may be hard to perform on the electrode, because the base plastic material on which these are printed may be susceptible to disintegration. Metal and conducting polymer nanowires are mainly prepared based on a template-directed electrochemical synthesis. One-dimensional nanomaterials such as nanowires and nanotubes are of vital importance in electrochemical biosensors due to their inherent anisotropies and efficient transport of electrons and excitons within the smallest dimension [55]. For example, carbon nanotubes are widely used in enzyme electrodes [56,57,58], which are a common component of electrochemical biosensors. Patolsky et al. [59] demonstrated the production of aligned reconstituted glucose oxidase with the help of single-wall CNTs that can be easily linked to the electrode surface. Enzyme electrodes combining dehydrogenase or oxidase enzyme that depend on the amperometric monitoring of liberated NADH or hydrogen are a challenge. The detection is usually effected as a result of the large overvoltage encountered during their oxidation. The electrodes modified by CNTs avoid this difficulty, addressing these overvoltage limitations [57]. Carbon nanotubes are used as molecular wires owing to their unique small size and desirable physical properties. Carbon nanotubes include single-wall carbon nanotubes, which can be viewed as molecular wires with all the atoms on the surface, produced as a result of rolling a single graphite sheet into a tube, and multi-wall CNTs. As CNTs possess many desirable structure-dependent mechanical and electronic properties, they can contribute to the possibility of manufacturing superior electrochemical biosensors. One-dimensional nanostructures including CNTs and semiconductor-or-conducting polymer nanowires are widely used in bioelectrochemical detection, raising the prospect of rapid and sensitive label-free bioelectronics detection [60]. However, one-dimensional CNTs have a random network, which may cause a lack of reproducibility in electrochemical sensing. More recently, to overcome the mentioned problem, the use of graphene, a two-dimensional carbon nanostructure, has been proposed. In 2011, a graphene-based biosensor system was used to detect pathogenic virus [61]. Using a speed vacuum concentrator combined with a thermal annealing process, a graphene oxide (GO) film was synthesized from a suspension, and then it is used as a working electrode, which showed great electron transfer property. Nanomaterials benefit a lot in bioaffinity electrochemical sensors. Nanoparticle-based amplification schemes assist in improving the sensitivity of bioelectronic assays significantly.

## 3. Application of Electrochemical Biosensors for Disease

A biomarker is a characteristic indicator of some biological, pathogenic process [11], or pharmacological response to therapeutic intervention, with some property that can be measured and evaluated objectively [60]. The use of an appropriate biomarker that makes the detection procedure highly sensitive and selective is of vital significance. In the detection of diseases, various strategies have developed based on specific biomarkers [4,62,63,64]. To our knowledge, antigens/antibodies, as well as DNA/RNA series [19], enzymes and other biomolecules (e.g., protein, fructosyl valine) [17] are the most commonly used [2]. In the following part, we will discuss each kind mentioned above, combining a variety of techniques used in electrochemical biosensors.

### 3.1. Antibody as Recognition Biomolecule

**Figure 6:**
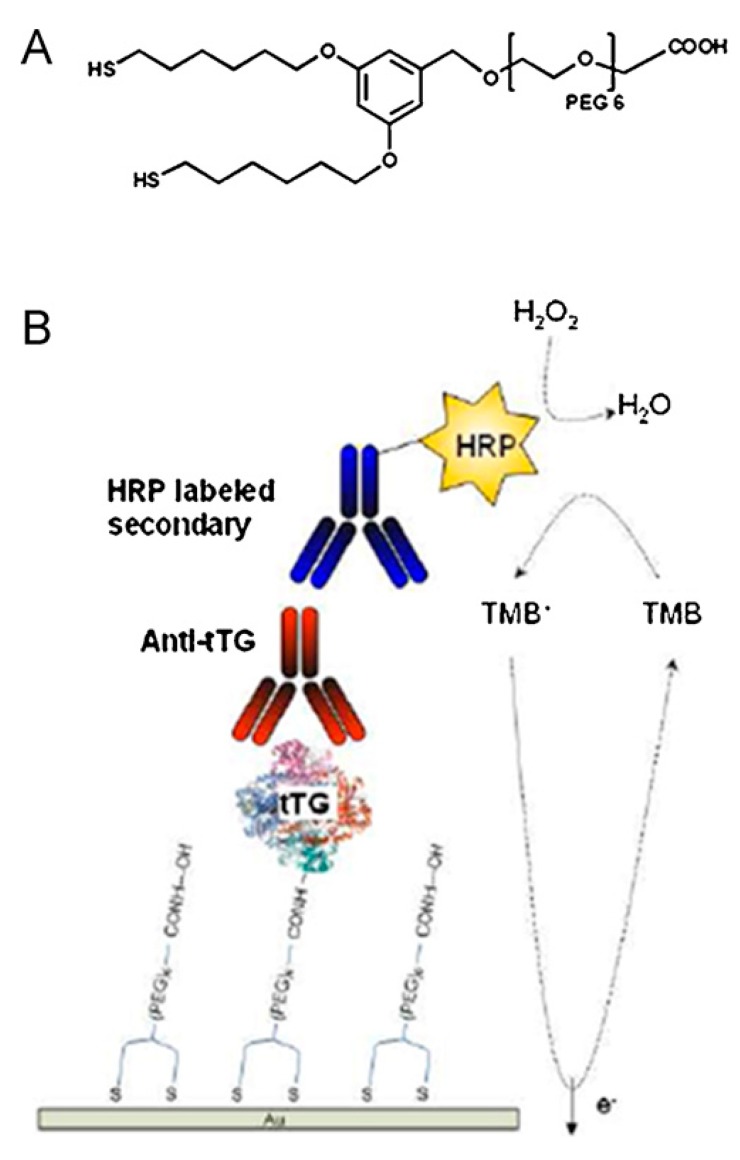
(**A**) (22-(3,5-bis((6 mercaptohexyl)oxy)phenyl)-3,6,9,12,15,18,21-heptaoxadocosanoic acid dithiol PEG-6 carboxylate (DT2); (**B**) Schematic of the electrochemical immunosensor assay architecture [70]. Copyright 2011. Reproduced with permission from Elsevier B.V.

The measurement of tumor markers in blood or serum can be utilized to screen for some diseases [65,66]. Some tumor markers are antigenic in essence, such as carcinoembryonic antigen, prostate specific antigen [67] and human chorionic gonadotropin. An electrochemical immunosensor is a typical device used for detection of these diseases, in which a sandwich immunoreaction is usually performed on an electrode surface (see Figure 6), including a tracer antibody, a capture antibody and the antigen. It depends on whether there an inflammatory response or antigen-antibody reaction, which serves as a characteristic marker exists. Dixit et al. proposed electrochemistry-based approaches for cancer diagnosis [68] with low cost, high sensitivity, automated multiplexed protein immunoassays. Similarly, Kadimisetty et al. [69] proposed automated multiplexed ELC immunoarrays for cancer biomarker proteins. Celiac disease is an autoimmune-mediated disorder, which has a strong genetic predisposition, and leads to the production of antibodies against gliadin and tissue transglutaminase. Human leukocyte antigen is the major factor causing the high risk that exists in affected individuals. The immunoglobulin IgA anti-human tissue transglutaminase (tTG) serves as an appropriate test. An electrochemical immunosensor could be applied to detect these anti-tissue transglutaminase antibodies [70]. Before this electrochemical method, the gold standard for celiac disease detection depended on a small intestinal biopsy. Experiments have proved the increasingly specific and sensitive characteristics of serological screening tests (e.g., enzyme linked immunosorbent assay (ELISA)) connected with autoantibodies, anti-gliadin and anti-tissue transgluminase [71]. In 2009, Pividori et al. [72] used an electrochemical immunosensing strategy to detect antibodies to tissue transglutaminase (tTG) in human serum, among various classes of antibodies in human serum toward tTG, the best results were achieved while anti-tTG antibodies were investigated. According to the result of processed serum samples, the sensitivity is 70%. Compared with traditional ELISA method, the specificity is 100%.

**Figure 7:**
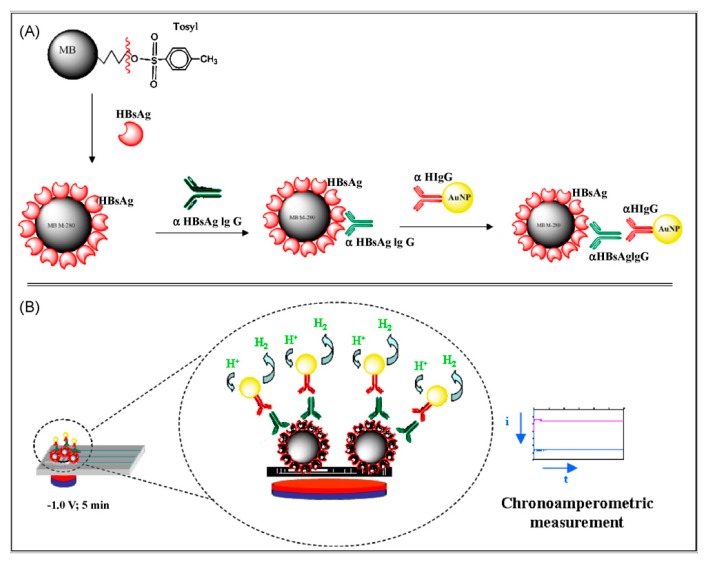
(**A**) Scheme of the experimental procedure performed. HBsAg are captured on the surface of magnetic beads, incubation with human serum that contains -HBsAg IgG antibodies and recognition with AuNPs conjugated with goat -human IgG antibodies; (**B**) The electrochemical detection procedure was based on the electrocatalytic hydrogen generation [6]. Copyright 2010. Reproduced with permission from Elsevier B.V.

Hepatitis B virus can be detected with electrochemical biosensors. This virus, which replicates primarily in the liver, but sometimes also in other organs or in lymphocytes, can easily cause a high human disease risk [73,74,75,76]. The chemical nature of the virus is a viral envelope protein called hepatitis B surface antigen. People who are exposed to the virus will accordingly show normally IgG antibodies against this antigen. These antibodies can be used as a recognition element. An electrochemical biosensor based on a sandwich immunoassay was used for rapid detection of anti-hepatitis B virus antibodies in human serum (see Figure 7) [6]. Magnetic beads were utilized as bioreaction platforms and Au nanoparticles (AuNPs) were used as electroactive labels. Through calculating the antibodies concentration in human sera samples, the biosensor allowed a detection limit of 3 mIU/mL. Alizadeh et al. [66] presented an electrochemical immunosensor using magnetic Fe_3_O_4_ nanoparticles (MNPs) as carriers for loading antibodies and methylene blue (MB) as electron transfer mediator. It was sensitive for the detection of hepatitis B virus surface antigen, using a Au sheet as working as electrode and hemin/G-quadruplex DNAzyme and gold nanoparticles as signal amplifier. The proposed immunosensor provided a novel platform for disease diagnosis. Incidentally, in recent years, magnetic beads have been utilized in the configuration of biosensing devices as bioreaction supports or carriers [63,77] owing to their ample surface area and the fact that they are biocompatible. Magnetic beads are controlled by a magnetic field, which can easily adjust their location and transport.

**Figure 8:**
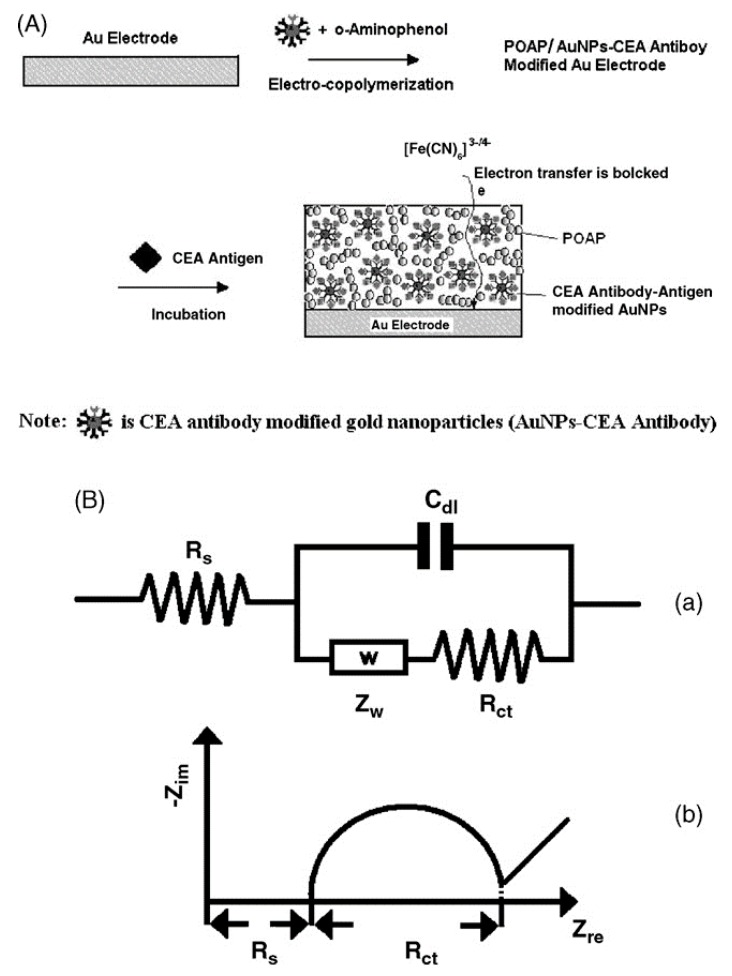
(**A**) Scheme for the electro-copolymerization of CEA antibody–AuNP bioconjugates with o-aminophenol on polycrystalline gold electrode and the formation of CEA antibody–antigen complexes; (**B**) Scheme for general equivalent circuit for impedance spectra analysis in the presence of a redox probe (**a**) and a typical Nyquist plot of an electrochemical impedance spectra (**b**) [85] Copyright 2006. Reproduced with permission from Elsevier B.V.

In addition, AuNPs with excellent properties can be not only used as electroactive labels [78], but they have also drawn attention for further enhancing the performance of gold nanoparticles label-based immunosensors. Biomolecule-NP hybrid systems, which combine both the recognition and catalytic properties of biomolecules and the catalytic properties of NP materials, with synergistic properties originating from the components of the hybrid composites [79], are a particular new approach. In 2010, Li et al. [80] proposed to use magnetic core/shell particles coated on a nanogold multilayer via self-assembly. In 2014, for the detection of carcinoembryonic antigen (CEA), which was considered as an oncofetal antigen expressed only in cancer patients, Huang et al. [81] used Ag/Au nanoparticles that were chemically functionalized to coat them on graphene. It’s worth noting that in this method Ag/Au nanoparticles were used as a signal amplifying factor. This Ag/Au nanostructure presents good electrochemical activity in the working potential range and it provides high current response. However, the time of analysis is a critical factor while considering the application in clinic analysis, and how to develop simpler and more effective detection methods is a future direction. Before that, Tang et al. [82] used a label-free electrochemical immunoassay electrode to detect CEA (see Figure 8). The CEA antibody was fixed covalently on glutathione (GSH) monolayer-modified AuNPs, and then the conjugate of CEA antibody and AuNPs were immobilized on electrode by electro-copolymerization with *o*-aminophenol (OAP); the detection limit was of 0.1 ng·mL^−1^ and a linear range of 0.5–20 ng·mL^−1^ was measured. It was proved successful in determining the CEA in human serum. Also, as this approach does not require sophisticated fabrication, and it is well suited for high-throughput biomedical sensing and application, the potential of this method is a simple and efficient strategy for immunoassays. Future work is to study whether it can be extended to the detection of other antigens or biocompounds, since the present assay system is only focused on the determination of the target antigen molecules. In addition, colloidal gold nanoparticles were presented to improve the reversibility of the electrochemical reaction. Wu et al. [83] detected CEA with a disposable immunosensor prepared by coating CEA/colloid Au/chitosan membrane at screen-printed carbon electrode (SPCE). The immunosensor integrated to a flow electrochemical system with an injection of sample, horseradish peroxidase (HRP)-labeled CEA antibody was utilized to trap the labeled antibody. The detection limit obtained was 0.22 ng·mL^−1^ (S/N = 3). Similarly, horseradish peroxidase (HRP) was used as label, encapsulated into nanogold hollow microspheres (GHS) in an in-situ amplified immunoassay for CEA detection [82]. The assay sensitivity was further increased by using HRP-GHS-anti-CEA with the detection limit of 1.5 pg·mL^−1^, compared with the previous HRP-anti-CEA method, the detection limit of that was 1.5 ng·mL^−1^. More recently, Xu et al. [84] detected CEA and α-fetoprotein (AFP) with an electrochemical immunosensor using metal ions tagged immunocolloidal gold nanocomposites as signal tags. A glassy carbon electrode (GCE) was modified with chitosan-AuNPs through glutaraldehyde. The detection limit was 4.6 pg·mL^−1^ for CEA and 3.1 pg·mL^−1^ for AFP. The linear response range of each one was within 0.1–50 ng·mL^−1^.

Liu et al. [86] developed a free-standing three-dimensional electrochemical immunosensor for the detection of CEA in real serum samples. Monolithic and macroporous graphene foam was grown by chemical vapor deposition, and served as the scaffold of the novel three-dimensional electrode. The macroporous structure ensured the efficient mass transport and made the bioaffinity ligands accessible, and the three-dimensional electrode with large surface area proved high density of the antibody. According to the results, this biosensor presents excellent performance in terms of detection range, sensitivity, lower detection limit and stability. The proposed immunosensor could be used as a versatile platform in clinical and biochemical analysis, and it gives a direction for detecting other proteins, by changing the respective antibodies. In 2016, lectin was used as molecular recognition element for discrimination of CEA. Zhao et al. [87] used both gold nanoparticles and enzymatic catalysis as dual signal amplification of electrochemical biosensor. The lectin-based biosensor array was successfully applied to evaluate the glycan.

**Figure 9:**
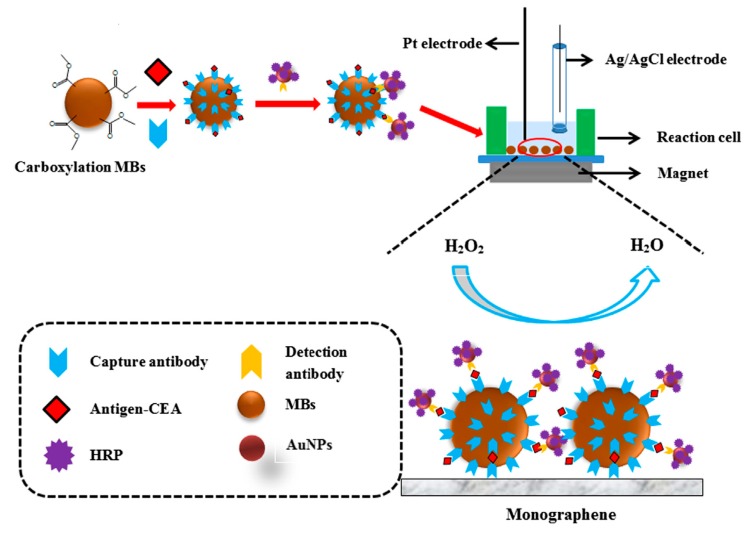
The preparation process of the multi-nanomaterial EC biosensor and the procedure of CEA detection [63]. Copyright 2013. Reproduced with permission from Elsevier B.V.

As disease treatment trials based on immunoassay are carried out successfully, there is a continual interest in electrochemical biosensors for more extensive applications with better performance [88,89,90]. To our knowledge, the detection limit is an important index for evaluating the performance of an electrochemical biosensor system. Other considerations such as immobilization method that affects the robustness of the link between the recognition element and the transducer, the number of active molecules after immobilization, the complexity of the procedure and even the relative materials are also of importance [25]. An immobilization by physical adsorption of tTG on graphite-epoxy composite (GEC)) electrodes was presented [71]. The tTG was from guinea pig liver. Through the test in human serum which contains the specific anti-tTG antibodies related to celiac disease, the result offered great potential for the analytical method. In 2011, Dulay et al. [70] used a self-assembled monolayer (SAM) based on thiols to immobilize tissue transglutaminase (tTG), the antigen for the immunorecognition of circulating autoantibodies, on a gold electrode surface. It is proved efficient, and it successfully maintained the tTG antigenicity. It was worth noting that it was reported capable of detecting human IgA antibodies at a very low concentration. This immunosensor allowed the estimation of anti-tTG IgG and IgA contents in real patients’ samples matching the performance of more laborious and time consuming procedures such as ELISA analysis. Clearly, dithiol-based surface chemistry modification has great potential to produce a rapid, reliable and low cost platforms for the detection of antibodies related to autoimmune diseases. In addition to the above, the presence of non-specific binding was another factor which affected the efficiency. The utilization of the polyethylene glycol moiety was proved effectively to eliminate non-specific binding of any sample matrix components. Complex matrices such as calibrator sera solution and real samples didn’t affect the performance of the electrodes. Much attention has been paid to signal amplification using nanomaterials. In 2008, Lin et al. [91] presented a biosensor integrating an immunochromatographic strip to detect PSA rapidly and sensitively, on which a sandwich immunoreaction is performed, with an electrochemical detector for signal transduction. This device employed nanoparticle (NP) labels made of CdSe@ZnS to amplify the signals. Through the detection in human serum, the approach was proved rapid due to the test strip format and highly sensitive due to the adoption of the NP-generated signal amplification. In 2016, another novel catalytic bionanolabel [92] was proposed for detection of *B. anthracis* Sap antigen. Bimetallic gold-palladium nanoparticles (Au-Pd NPs) were grown in situ on a boron nitride nanosheet (BNNS) which has been modified by diallyldimethylammonium chloride, and then the Au-Pd NPs@BNNSs conjugated with the mouse anti-*B. anthracis* Sap antibodies (A$b_{2}$). This conjugate bionanolabel exhibited high catalytic activity and opened a new direction for the detection of different biological warfare agents and their markers in different matrices. In addition, the utilization of graphene (GR) nanomaterial has been focused on electron transfer. GR is an excellent nanomaterial with two-dimensional sheets of s$p^{2}$-hybridized C atoms in hexagonal configuration [92]. In 2014, Jin et al. [63] optimized an electrochemical biosensor which is based on a graphene (GR) platform (see Figure 9) grown on 25 μm thick Cu through chemical vapor deposition (CVD) for detection of CEA. The proposed biosensor was associated with magnetic beads and enzyme-labeled antibody-gold nanoparticle bioconjugate. The magnetic beads were coated with capture antibodies and then attached to the graphene platform by an external magnetic field, and in this way, the reduction of the conductivity of graphene was prevented. The multi-nanomaterial was used to accelerate the electron transport procedure. Moreover, the multi-nanomaterial electrode exhibited a high GR electron transfer rate, and amplified the electro-signal produced by the MBs–AuNPs. The obtained results indicate a rapid response time and recovery time that are shorter than possible with traditional strategies.

An immunosensor based on three different generations of ferrocene (Fc) cored polyamidiamine dendrimers gold electrode was used to detect prostate antigen (PSA) [90]. To fabricate the electrode, the self-assembled monolayer (SAM) principle was used, and cysteamine (Cys) was covalently fixed on the Au electrode surface. The monoclonal antibody of PSA was immobilized by covalence. The authors used differential pulse voltammetry (DPV) to quantify the PSA levels, and the results showed a very low detection limit of 0.001 ng·mL^−1^, demonstrating the performance of the proposed method was excellent. In addition, Rahi et al. [93] used gold nanospears that had been electrodeposited as a transducer to immobilize an aptamer of PSA. Through the application for detection of PSA in blood serum samples of both health individuals and patients, the linear concentration range of aptasensor was proved to be 0.125–200 ng·mL^−1^.

**Figure 10:**
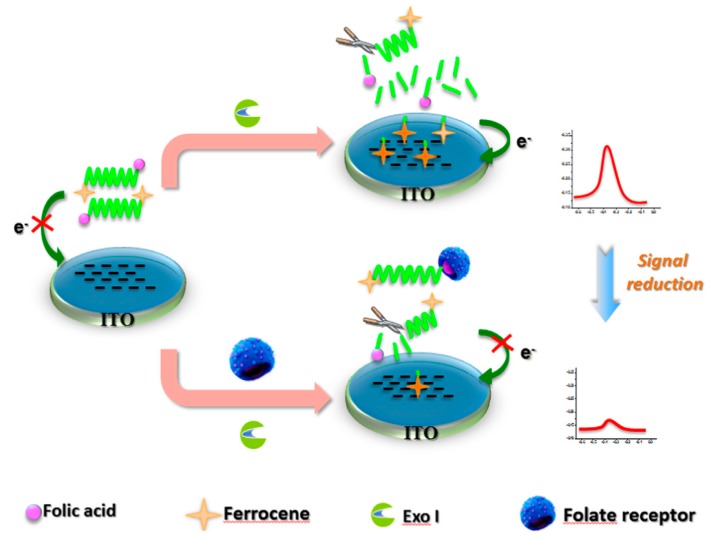
The principle of electrochemical biosensor for FR determination based on the immobilization free and terminal protection of small molecule linked DNA [95] Copyright 2016. Reproduced with permission from Elsevier B.V.

As another instance, dengue virus is an etiologic agent of dengue disease and it has been one of the most important emerging infectious pathologies [94]. In 2013, for the weak molecular bonding between the dengue virus (DV) and its receptor CLEC5A, Tung et al. [52] developed a method using a nanostructured electrochemical biosensor adopting gold nanoparticles (GNPs) uniformly deposited on a nanohemisphere array as sensing electrode, which proved highly sensitive. Up to then, the proposed approach was a highly promising tool for screening the actual binding site of protein-glycol conjugation. For detection of folate receptor over expressed in vast quantity of cancerous cells frequently, Ni et al. [95] proposed an electrochemical FR biosensor in cancer cells (see Figure 10). It adopted homogeneous indium tin oxide (ITO)-based electrochemical detection without immobilization. With the selectivity of terminal protection of small molecule linked DNA, this biosensor exhibited a wide linear range from 10 fM to 10 nM and a low detection limit with 3.8 fM (S/N = 3). In 2016, a new catalytic bionanolabel was designed for specific detection of *B. anthracis* Sap antigen. Sap referred to surface array protein which could be as a unique biomarker for *B. anthiracis*, the causative agent of the bioterrorism agent called anthrax. Sharma et al. [92] used Au-Pd nanoparticles@BNNSs nanohybrid as bionanolabels. The result indicated a fast and unique method of the specific detection of *B. anthracis* bacteria.

### 3.2. DNA/microRNA as Biomolecule

Due to the specific characteristics of the hybridization process between short DNA probes and target DNA (or RNA) sequences, genetic screening and detection of human nucleic acid sequences have been increasingly important for the diagnoses of various diseases [96,97]. Routinely, DNA-based electrochemical biosensors take advantage of nanoscale interactions between the target in solution [98], the recognition layer and a solid electrode surface modified with silicon [99], gold or carbon graphene [100]. Voltammetry techniques are typically used (e.g., differential pulse voltammetry (DPV), square wave voltammetry (SWV), electrochemical impedance spectroscopy (EIS) [3] and cyclic voltammetry (CV) [101]). In the past years, an impressive number of electrochemical DNA biosensors have appeared for DNA/RNA sequence analysis such as the detection of human immunodeficiency virus type1(HIV-1) short DNA sequence , hepatitis B virus [101], influenza viruses (e.g., H1N1, H3N2) [34], oral cancer [102], etc.) In this method, the detection may be realized in molecular level [103]. In 1996, Joseph et al. [103] applied an electrochemical biosensor to detect short DNA sequences related to human immunodeficiency virus type 1(HIV-1), avoiding the use of radioisotopes involved in traditional solutions. Generally, polymerase chain reaction (PCR) amplification is used to obtain DNA sequence in voltammetry technique [33,35,104]. Azek et al. [76] presented a disposable electrochemical DNA biosensor to detect human cytomegalovirus (HCMV)-related DNA sequences, where a detection limit of 0.6 amol·mL^−1^ of HCMV-amplified DNA fragment was obtained. The method was 23,000-fold more sensitive than the electrophoresis technique while 83-fold more sensitive than the colorimetric hybridization assay in a microtiter plate. Kara et al. [35] used an electrochemical biosensor combined with the DPV technique to detect DNA sequences related to HSV and discriminate between HSV Type1 and Type2 virus from PCR amplified real samples. MB was used as indicator. Another approach for the detection of influenza B virus from PCR samples was used by Aydinlik et al. [88], who introduced a method utilizing AuNPs. Meldola’s Blue (MDB) was used as intercalator label. The technique may realize the electrochemical detection of influenza B virus based on DNA hybridization. Through the detection of real samples, the results obtained indicats that the utility of AuNPs could enhance the electrode surface area. Specially, if the PCR amplicons are unpurified, there is a reference method since Ahmed et al. [105] introduced a biosensor using disposable electrochemical printed (DEP) chips connected with the redox active molecule Hoechst 33,258 [2′-(4-hydroxyphenyl)-5-(4-methyl-1-piperazinyl)-2,5′-bi(1*H*-benzimidazole)] to detect single nucleotide nucleotide (SNPs) from unpurified PCR amplicons.

**Figure 11:**
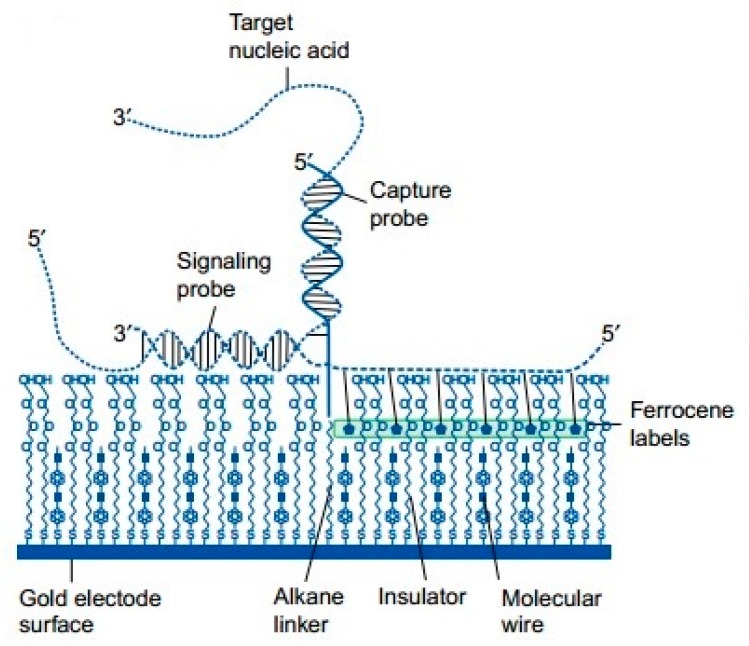
Scheme of the sandwich assay for electronic detection of nucleic acids. The gold electrode was coated with a SAM including (1) DNA-alkanethiols that contain the capture probe sequence; (2) molecular wires, which provide a pathway for electron transfer between the Fc and the gold in response to potential changes at the electrode; and (3) alkanethiols terminated in polyethylene glycol insulator, which serve as insulators to block access of redox species in solution to the electrode, including free signaling probes. A target nucleic acid is shown annealed to a capture probe and a Fc-labeled signaling probe [106]. Copyright Reproduced with permission from American Society for Investigative Pathology and the Association for Molecular Pathology.

**Figure 12:**
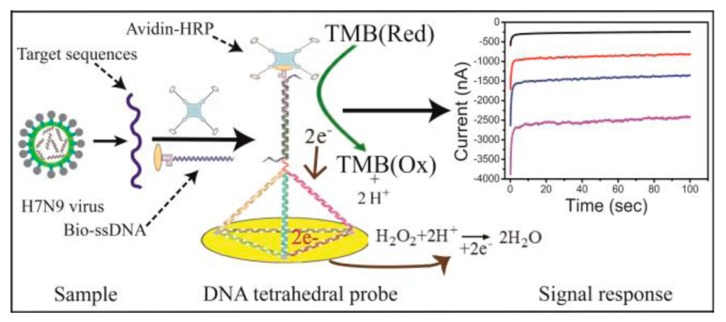
Diagram of DNA tetrahedral probe [34]. Copyright 2015. Reproduced with permission from American Chemical Society.

There are two types of DNA sequence, including single-stranded DNA (ssDNA) and double-stranded DNA (dsDNA). In a typical configuration, an ssDNA probe sequence is immobilized within the recognition layer, where base-pairing interactions recruit the target DNA to the surface. Double-stranded DNA is formed by two single strands (ss-DNA) bonding to each other [106] (see Figure 11). In 2010, Pournaghi et al. [107] developed an electrochemical biosensor for direct detection and discrimination of dsDNA corresponding to hepatitis C virus genotype 3a to study whether the developed biosensor could respond selectively to the dsDNA target. The electrode of the sensor has been modified with 6-mercapto-1-hexanol and a self-assembled monolayer (SAM) of 14-mer peptide nucleic acid probe (PNA probe) that was related to the hepatitis C virus genotype 3a core/E1 region. The authors carried out experiments with a detection limit was 1.8 pM in phosphate buffer solution at pH 7.0, showing the possibility of monitoring the hybridization of the PNA probe with the target ds-DNA. In 2015, Benvidi et al. [3] presented a reproducible label-free electrochemical ssDNA biosensor using [Fe(CN)6]-3/-4 as redox couple with a gold electrode to detect breast cancer. Self-assembled monolayers (SAMs) of thiolated ssDNA or BRCA1 5382 insC mutation detection were immobilized on the electrode. The authors adopted electrochemical impedance spectroscopy (EIS), cyclic voltammetry (CV) and DPV respectively to characterize the electrode. Measurements of complementary DNA strains done by EIS method were excellent, with a wider dynamic range and a lower detection limit at 4.6 × 10^−20^ for the target DNA as compared to other methods. In earlier 2003, through the comparison between the interactions of Fc+ with HBV ssDNA and HBV dsDNA immobilized on TGA monolayer, the presented strategy using ssDNA was indicated highly sensitive and suggested more convenient for monitoring DNA hybridization [33]. In 2015, a novel DNA tetrahedral DNA nanostructure-based biosensor (see Figure 12) was reported to detect avian influenza A (H7N9) virus [34]. The DNA tetrahedral probe was immobilized on a gold electrode surface by self-assembly, between three thiolated nucleotide sequences and a longer nucleotide sequence including complementary DNA and hybridized with the target DNA. From the result, this proposed electrochemical biosensor could not only recognize the target DNA fragment of H7N9 from other types of influenza viruses (e.g., H1N1, H3N2), but also from mismatches of oligonucleotides. Moreover, the detection limit was found to reach a magnitude of 100 fM for the target nucleotide sequence, improving the detection performance of electrochemical biosensor compared with general ssDNA-based biosensors. It was the first time that the utility of the DNA tetrahedral was reported, showing the great potential of DNA tetrahedral as a probe applied for detection of H7N9 virus. A similar DNA tetrahedral DNA nanostructure probe combined with hybridization chain reaction (HCR) amplification was used for sensitive detection of microRNA [34]. The tetrahedron was synthesized in one step which avoided complicated fabrication steps. The detection limit was found to be 100 aM for DNA and 10 aM for microRNA, corresponding to 600 microRNAs in a 100 μL sample. This trial proved the effect of DNA tetrahedron for detection of both DNA and microRNA.

**Figure 13:**
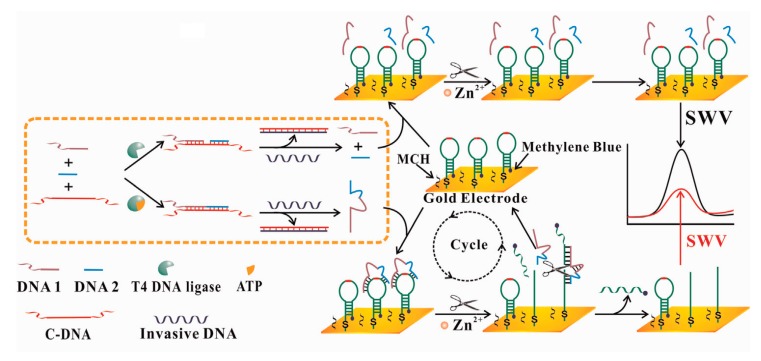
Scheme of the electrochemical sensing system based on the dual strategy of ATP-dependent enzymatic ligation reaction and cyclic amplification based on self-cleaving DNAzyme [89] Copyright 2014. Reproduced with permission from Elsevier B.V.

In 2008, a label-free hybridization biosensor using 2,9-dimethy1-1,10-phenantroline cobalt [Co(dmp)(H_2_O)(NO_3_)2] as indicator was applied to detect hepatitis B virus [108], whereby a 21-mer probe DNA was immobilized onto GCE and then hybridized with target DNA. Cyclic CV and DPV were used for electrochemical detection. The results exhibited a detection limit of 1.94 × 10^−8^ M (S/N = 3) and the current respond had a linear relationship with the concentration of target DNA ranging from 3.96 × 10^−7^ to 1.32 × 10^−6^, demonstrating good selectivity by detecting the three-base mismatch sequence ssDNA. To our knowledge, the options used in previous studies could be daunomycin [104], ferrocenium hexafluorophosphate (FcPF6) [104], Meldola Blue (MDB) [35], MB [109], etc. MB was used as hybridization indicator to detect DNA sequence that relates to Chronic Myelogenous Leukemia (CML, Type b3a2) [36]. The authors observed a peak current increase for MB upon the hybridization of immobilized ssDNA with cDNA in the solution, finding that under optimal conditions, the biosensor has a good calibration with DNA sequence detection limit of 5.9 × 10^−7^ M. Similarly, a label-free biosensor using MB as redox indicator [7] was used for microRNA related to breast cancer. Combined with the DPV technique, which was used to record the oxidation peak current of MB under optimal conditions after hybridization of ssDNA probe and target microRNA, an increase of peak current was observed. The response was thus calculated with the microRNA range from 0.1 to 500.0 pM, moreover, the detection limit was of 84.3 fM. Guo et al. [110] proposed a DNA electrochemical biosensor in which cationic polymer chitosan was used to modify a carbon paste electrode (CCPE) for detection of short DNA sequences associated with the hepatitis B virus (HBV). MB was used as indicator observed upon hybridization of probe with the target and DPV was used for peak current measurement. A similar method was also used by Li et al. [24]. This method did not need use mercapto-DNA biotin-DNA, so it reduced the detection cost. For HBV detection, Ye et al. [33] designed covalently immobilized HBV ssDNA on gold electrode via a carboxylate ester as a linkage between the 3′-hydroxy end of DNA and the carboxyl groups of a thioglycolic acid (TGA) self-assembled monolayer and used ferrocenium hexafluorophosphate (FcP$F_{6}$) as indicator. This approach provides a DNA detection of 1 × 10^4^ copies of original genomic HBV DNA. Further, in a procedure of fabricating DNA electrochemical biosensor [24], the covalent immobilization of target ssDNA implemented on Au electrode which had been functionalized with two siloxanes, 3-mercaptopropyltrimethoxysiloxane (MPTMS) and 3-glycidoxypropyltrimethoxysiloxane (GPTMS) as precursors to form a self-assembly sol-gel film, the thiol group of MPTMS allowed assembly of MPTMS sol-gel on electrode surface. The linear range was from 2.51 × 10^−9^ to 5.02 × 10^−7^ M and the detection limit was 8.57 × 10^−10^. Further, bifunctional method is most effective for detection. Chung et al. [108] developed an electrochemical DNA biosensor with a probe DNA avidin-biotin conjugation and a GCE modified with avidin for the detection of influenza virus (Type A). In 2014, Lu et al. [89] developed an electrochemical biosensor based on a dual strategy which combines the adenosine triphosphate (ATP)-dependent enzymatic ligation reaction with self-cleaving DNAzyme-amplified electrochemical detection (see Figure 13). The presented biosensing platform could specifically distinguish the target biological molecule ATP from its six analogues due to the highly specific cofactor-dependence of T4 DNA ligase. Other diseases such as salmonella [111], breast cancer and lung cancer [62] were detected with electrochemical biosensors. Ma et al. [111] used graphene oxide and gold nanoparticles to modify a GCE of electrochemical biosensor for detection of ssDNA related to *Salmonella*. The thiolated *Salmonella* aptamer ssDNA sequence was immobilized on the electrode, more incubated *Salmonella* may lead to the decrease of current, resulting in the increase of impendence. According to the detection, the limit was proved excellently low with 3 cfu/mL. An immobilization-free electrochemical DNA biosensor was presented [102] to detect oral cancer, it was more rapid than traditional electrochemical DNA biosensors, the assay owns the merits performed in a homogeneous solution but also exhibits high distinction ability to single-base mismatch, double-bases mismatch and non-complementary DNA sequences.

**Figure 14:**
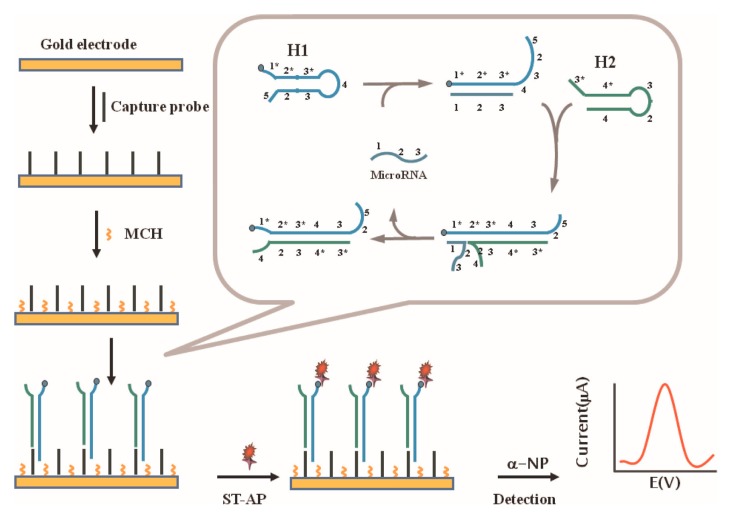
Principle of miRNA electrochemical detection based on mismatched catalytic hairpin assembly amplification [114]. Copyright 2015. Reproduced with permission from Elsevier B.V.

**Figure 15:**
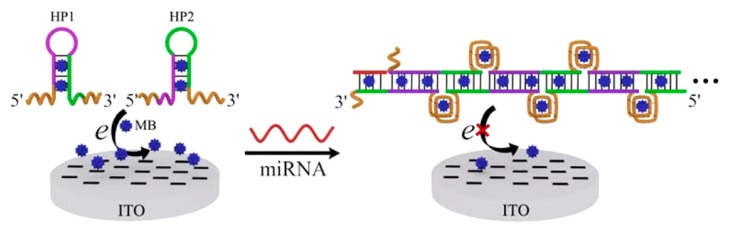
Mechanism of the label-free and enzyme-free homogeneous electrochemical strategy based on HCR amplification for miRNA Assay [116] Copyright 2015. Reproduced with permission from American Chemical Society.

Other than DNA, detection of microRNA also plays critical role in various diseases, particularly in early clinical diagnosis. MicroRNAs may be as promising biomarkers for cancers [112] (stomach, prostate, lung, pancreas, colon, etc.) and other diseases (e.g., heart diseases, chronic lymphocytic leukemia, diabetes). However, there are only a few microRNAs with short length in cells, the sensitive detection of microRNA is a challenge. An integrated minipotentiostat was used [113] to develop the nucleic acid-based electrochemical microfluidic biosensor. It measures and stores the current, and interdigitated ultra-microelectrode array for the quantification of RNA, demonstrating the performance through the detection result of dengue virus RNA. Compared with the standard method, the minipotentiostat performance was comparable in signal strength, meanwhile achieving a lower detection limit. However, the minipotentiostat responded slower to higher concentrations of virus than a bench-top system. Zhang et al. [114] developed a simple electrochemical biosensor for microRNA detection using mismatched catalytic hairpin assembly (CHA) which could decrease the non-specific CHA products that affected background signal (see Figure 14). Through discrimination results of target miRNA from mismatched miRNA and the determination of miRNA spiked into human total RNA samples, the detection limit for target miRNA was 0.6 pM (S/N¼3) with a linear range from 1 pM to 25 nM. In 2013, a label-free microRNA biosensor was developed [115], Ren et al. presented an isothermal signal amplification strategy based on the hybridization between target microRNA and the remaining CPs on the electrode. The procedure was much simplified, and it suggested that the proposed biosensor can substantially be upscaled, by adopting the complementary metal-oxide semiconductor technology for biosensor/array fabrication, and by replacing the manual operation with a microfluidic cartridge. It improved the suitability in direct profiling miRNA expressions with minimal or no sample pretreatments. Therefore, it may be an attractive candidate for the development of a simple, robust, and sensitive miRNA expression profiling platform Mandli for uses at point-of-care. Similar approach in 2015 was reported [116], a homogeneous electrochemical biosensing strategy was used for detection of microRNA (see Figure 15).

This strategy was label-free and enzyme-free. It was based on hybridization chain reaction (HCR) amplification. Using this “signal-off” mode, the strategy successfully distinguished the microRNA, including the single-base mismatched microRNA. For more sensitive and specific detection, magnetic-controllable RNA biosensor for detection of microRNA related to oral cancer was presented [112], the electrically electrode includes the advantages of both heated electrode and magnetic electrode. The detection limit was 2.2 × 10^−9^ M with a recovery of 93–108% and a RSD < 6. More recently, Mandli et al. [117] reported an electrochemical miRNA-21 biosensor which adopted sandwich type hybridization assay performed on AuNPs modified pencil electrode (PGE) combining with enzyme enhancement for signal amplification. The thiol-terminated capture probe 1 interacted with Au and was then immobilized onto the electrode, then hybridized with the first part of the target microRNA-21. Meanwhile, the other part hybridized with a biotinylated probe 2, the strong interaction allows good capture of target. It’s worth noting that the strong interaction between thiol conjugated capture probe and AuNPs allows the good capture of miRNA-21 target, and that each SA-ALP can catalyze the production of a large amount of electrochemically active molecules. The established miRNA assay exhibited a high sensitivity, and a low detection limit was carried out which was 100 pM (10 fmol in 100 μM in 45 min).

### 3.3. Enzyme as Biomolecules

In some cases, enzymes may provide valuable information for disease diagnosis. The main detection strategies employed for enzyme determination include colorimetric [118], chemiluminescense [119], chromatography [120] and electrochemical techniques [116]. These strategies usually require complex reagents that need lots of time, meanwhile, they involve many operation procedures. Thus, there is a need for a method which could work in situ, with reduced time and money cost. Several biosensors for enzyme determination were proved to be a promising alternative. Electrochemical enzymatic biosensors have been developed in recent years, the development of which goes back over several decades.

In 1967, an enzymatic biosensor was reported for the first time, in which glucose oxidase enzyme in a polyacrylamide gel was immobilized on the electrode. Owing to the high biocatalytic activity and specificity of enzymes [121], electrochemical biosensors primarily used enzymes as biosensing devices. Alanine aminotransferase (ALT) plays an important role in the diagnosis of liver disease, residing within the cells of the liver while in normal conditions but spilling into the blood stream while in injured condition. Chang et al. [122] presented an electrochemical biosensor based on a palladium electrode modified with glutamate oxidase to monitor ALT. Earlier more than this assay, He et al. [123] adopted a kinetic DPV to determine the activity of ALT in human serum, based on a gold electrode without modification. Later in 2009, Jamal et al. [124] developed a dual working electrochemical biosensor which was based on a platinum electrode modified with glutamate oxidase. The activity of ALT over its both normal and elevated physiological range was developed with the detection of L-glutamate at the electrode. The sensitivity of the device and a linear range were found with amperometry under specific conditions. It was proved to be mediated and stable with fast response time characteristics. To improve the sensitivity and selectivity of a system, choosing appropriate materials and structure of electrode may be of use. In 2008, Fang et al. [125] proposed a single-use, disposable FV electrochemical biosensor, the prototype of which was modified with iridium, combining thick film screen-printing technique. FV referred to fructosyl valine, which was important for the long-term management of diabetic patients, as it was modeled for the glycosylated hemoglobin (HbA_1c) detection, whereas the glycosylated hemoglobin detection played an important role in monitoring diabetic patients [126]. The enzyme fructosyl amine oxidase from recombinant *Escherichia coli* was immobilized on the working electrode of the biosensor prototype. The result shows that the biosensor operates at ambient temperature and required 3 μL of sample volume, meeting with the requirement including small sample volume and an appropriate temperature. Particularly, a bifunctional aptamer-based electrochemical biosensor was developed [127] to detect thrombin and adenosine without label, thrombin aptamer was firstly immobilized on gold electrode surface by self-assembly, and then it was hybridized with adenosine aptamer. Researchers applied the presented biosensor to human plasma samples analysis. The detection limit was 3 nM for thrombin and 10 nM for adenosine, respectively. The assay was linear in the ranges from 6–60 nM for thrombin and from 10 to 1000 nm for adenosine, in addition, interferents such as bovine plasma albumin (BSA) and lysozyme, or uridine and guanosine, had no influence on the biosensor performance, and this system was proved well selective.

**Figure 16:**
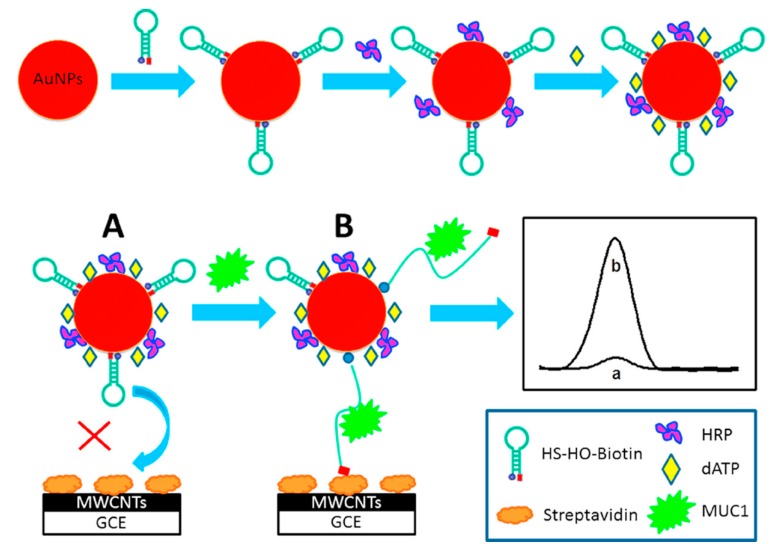
The scheme of MUC1 detection with the HO switch. (**A**) The biotin is shielded and thus inaccessible to the streptavidin in the absence of MUC1. Then, a very limited background current (inset) was observed (curve a); (**B**) The disruption of the stem-loop makes the biotin exposed upon target binding. Then, the biotin along with the dually labelled aptamers is easily captured by the streptavidin-modified electrode (curve b) [129]. Copyright 2013. Reproduced with permission from Elsevier B.V.

An electrochemical biosensor based on prostate-specific antigen was used for the screening of prostate cancer [5], however, researchers have found that PSA in serum is not specific to prostate cancer. One of the major limitations of PSA screening is that serum PSA can be elevated in patients with other common benign conditions [5]. Moreover, it is unable to distinguish between aggressive and indolent cancers. In 2012, Lin et al. [5] developed an electrochemical biosensor adopting α-methylacyl-CoA racemase (AMACR) as biomarker to detect prostate cancer. The nanoparticle metallic catalyst electrochemical biosensor was used to deal with plasma samples from 24 men, and successfully, distinguished healthy men, men with high grade prostate intraepithelial neoplasia and men with biopsy-proven prostate cancer with high accuracy (100%). Before that, AMACR was detected in 137 patients with prostate cancer and achieved an accuracy of 100%. However, the drawback is that the sample size is still limited at this stage. To make the technique work effectively, many requirements should be met, including the reaction mechanism, the presence of potential interfering species, the design of the biosensor and the electrode material that were well defined. Moreover, the proposed biosensor requires about forty hours of incubation time, the length of which should be a focus in future studies. Multiple tumor markers may provide useful and reliable information for clinical diagnosis, thus, it has attracted attention [128]. Hu et al. [129] reported a platform based on a hairpin oligonucleotide (HO) switch connected with AuNPs and enzyme signal amplification (see Figure 16). AuNPs were used as labels and foundation which carries the HO aptamer and the enzyme, forming a tracing tag to ultrasensitively detect mucin 1 protein (MUC1), a well-known tumor marker existing in various malignant tumors. This biosensor was proved to possess a good linear correlation range from 8.8 nM to 353.3 nM and a low detection limit of 2.2 nM for MUC1, providing an approach for the detection of a variety of diseases. Similarly, in 2014, Xu et al. [84] detected CEA and α-fetoprotein (AFP) with an electrochemical immunosensor using metal ion- tagged immunocolloidal gold nanocomposites as signal tags. The GCE was modified with chitosan-AuNPs through glutaraldehyde. The detection limit was 4.6 pg·mL^−1^. for CEA and 3.1 pg·mL^−1^ for AFP. The linear response range of each one was within 0.1–50 ng·mL^−1^.

### 3.4. Other Biomolecules

**Figure 17:**
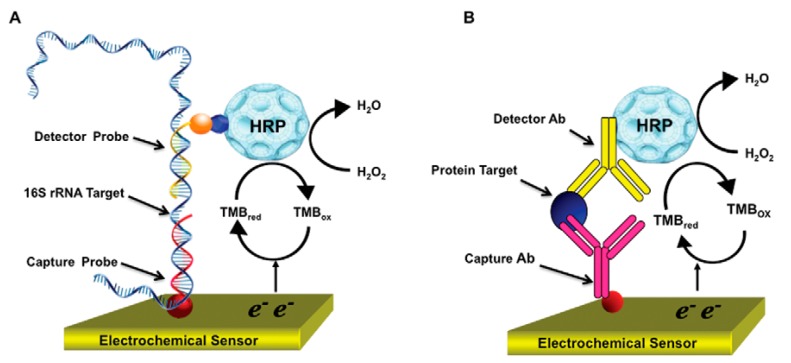
Illustration of urine-based detection of nucleic acids and proteins. (**A**) Schematic of pathogen identification based on sandwich hybridization of bacterial 16S rRNA with capture and detector oligonucleotide probes; (**B**) Schematic of immunoassay based on sandwich detection host urinary protein with capture and detector antibodies [133].

New kinds of recognition elements have emerged. Techniques to select aptamers known as chemical antibodies have been developed. Aptamers offer advantages including ease of synthesis, easy labeling and good stability. The affinity of aptamers involves targets such as proteins, organic molecules, metal ions and even whole cells [130,131]. Proteins, for instance, are intimately associated with current biological status in constituting the final or active form of the former. Thus, proteins as biomarkers in biological media hold great promise in disease-related detection. Hansen et al. [132] used a nanoparticle-based electrochemical sensor for the detection of several protein targets. The coupling of aptamers with the coding and amplification features of inorganic nanocrystals was shown for the first time. In 2011, Mohan et al. [133] presented an integrated electrochemical biosensor capable of detecting urinary biomarkers which could assist in improving the effectiveness of clinical disease management, indicating that in terms of diagnosis of urinary tract infection, pathogen identification combining with quantitative detection of lactoferrin may provide vital information (see Figure 17). Another electrochemical biosensor array with alkanethiolate self-assembled monolayer (SAM) was used for direct detection of the urinary tract infection (UTI) biomarker lactoferrin from infected clinical samples [134]. A sandwich amperometric immunoassay was used to detect lactoferrin from urine and detection limit of 145 pg·mL^−1^ was obtained. Lactoferrin was validated as a biomarker of pyuria (presence of white blood cells in urine), an important hallmark of UTI, in 111 patient-derived urine samples. An electrochemical biosensor based on biomimetic material for myoglobin detection was presented [135], in which a new reusable molecularly imprinted polymer (MIP) material assembled on a polymer layer that were casted on the gold area of a screen printed electrode. Combined with EIS and the SWV technique, this biosensor showed great robustness, reusability and stability. As another example, vascular endothelial growth factor (VEGF) is a protein that could be as a serum biomarker associated with various diseases. It may stimulate the growth, survival and proliferation of vascular endothelial cells [136]. In 2010, Zhao et al. [137] presented a folding-based electrochemical aptasensor for the detection of VEGF in complex biological samples (e.g., blood serum, whole blood). Owing to the electrochemical signaling mechanism linked to a target-induced conformational change of the aptamer probe, the biosensor was highly selective with less non-specific binding. Further, as the sensor was in fully covalent architecture, it is regenerable and reusable.

Other biomolecules, such as cholesterol in blood are important, as its high accumulation in blood is strongly associated with many diseases, and it is a major component of proteins in cell plasma membranes involved in signal transmission [138], and thus, it could be biomarker. Aravamudhan et al. [139] estimated cholesterol in blood using a biosensor that used aligned Au nanowires in a micro-fluidic platform. The biosensor showed a linear relationship between cholesterol level and current response, then sensitivity was 0.85 nA/μM. Chloramphenicol (CAP) is a broad spectrum antibiotic which is important for clinical analysis. In 2013, a facile and stable electrochemical biosensor for detection of CAP, exploring its direct electron transfer in in-vitro model was developed. Yadav et al. [140] used an aptamer to bind with CAP and adopted a quartz crystal microbalance (QCM) to ensure the interaction between the aptamer and the polymer film. Cyclic voltammetry (CV) combined with square wave voltammetry (SWV) were also used for the detection. The detection limit was as low as 0.02 nM which indicated the sensitivity of the present sensor. Most aptamer-based sensors are only able to detect one target. Deng et al. [141] made efforts to develop a bifunctional electrochemical biosensor for parallel detection of small molecule (adenosine) or protein (lysozyme). The system possess two aptamer units, one of which for small molecule (adenosine) and the other of which for protein (lysozyme).

## 4. Conclusions and Outlook

In the past few decades, the development of sensors remains an active area of analytical chemistry research as evidenced by the amount of papers published. Meanwhile, biosensors for detection of various diseases have aroused a great deal of interest. The commercialization of biosensors and their applications to clinical analysis have been reviewed, and the important role of biosensors in clinical tests has been demonstrated in previous reviews. In this review, however, we emphasize the impact of electrochemical biosensors, a subclass of biosensors, for disease-related analysis. Their unique characteristic attributes were shows, and various novel functionalized electrochemical biosensors were discussed. As shows above, the focus has been placed on the electrode fabrication, the immobilization techniques, measurement tools and relative materials that are used as analytical tools for disease-related detection.

Antibodies, DNA, microRNA, enzymes and other biomolecules have been widely used for accurate detection of a variety of diseases such as hepatitis B, breast cancer and lung cancer. Both single-stranded DNA (ssDNA) and double-stranded DNA (dsDNA) could be used as target and ssDNA is considered more convenient for monitoring DNA hybridization. MicroRNA may also be as a promising biomarker and strategies for the highly sensitive detection of microRNA have been developed. Several immobilization methods used to immobilize biomolecules onto electrode surfaces were presented. Various substances such as MB, daunomycin and ferrocenium have been utilized as indicators for the observation of the reaction after the hybridization reaction occurs on the electrode surface. Moreover, the measurement techniques including DPV, SWV, EIS and CV used to monitor the electrode as well as the nanomaterials, magnetic beads that are used for fabrication of electrochemical biosensors are also mentioned. Researchers have found electrochemical biosensors to be simple, sensitive, selective and rapid. Meanwhile, they save costs compared with previous methods such as Enzyme-Linked Immunosorbent Assay (ELISA) and Microparticle Enzyme Immunoassay (MEIA). It is demonstrated a promising analytical method reaching the requirements for amperometric measurement and with potential as an analysis biosensor.

Electrochemical biosensors have been proved to offer advantages such as simplicity and cost-effectivity, and they exhibit good sensitivity and selectivity under optimized conditions. In some cases, a very low concentration of samples is needed. The field of electrochemical biosensors has undergone a steady improvement in the past few decades. The detection limits have been lowered, and the linear response range has been enlarged as many efforts have been devoted to the improvement of electrodes, signal amplifiers and indicator labels. Indeed, modifying the electrode with various coatings and adopting advanced nanomaterials and proper indicators may influence the sensor performance. However, though a low detection limit has been achieved, the minipotentiostat may respond slower at higher concentration in some cases. On the other hand, a larger number of clinical samples is needed for processing. There are not many strategies directed toward reducing sample volumes or time of analysis in the literature. Thus, there is still some way to go before electrochemical biosensors are widely used in clinical laboratory instead of in the research laboratory. A clear direction of future work is still in molecular diagnostics for achievement of higher sensitivity and stability. Meanwhile, analytical validation by processing a higher number of clinical samples coming from patients is needed. Some parameters need to be considered as well such as the nature of the antigen, enzyme, protein or other biomolecules, as well as the oriented immobilization. Thus, the choice of recognition element as well as optimized immobilization strategies need to be researched in future studies.

## Supplementary Materials

The Supplementary Materials are available online at http://www.mdpi.com/1424-8220/17/10/2375/s1.
