# Supplementary material for: Disease-Related Detection with Electrochemical Biosensors: A Review
Source: Sensors (Basel). 2017 Oct 17;17(10):2375. doi: 10.3390/s17102375 (PMC5676665; doi:10.3390/s17102375)
Supplement: Supplementary file 1 [file sensors-17-02375-s001.pdf]

Supporting Information

Disease-related Detection with Electrochemical Biosensors: A Review

Ying Huang[1], Jin Xu[1], Jun Jie Liu[1], Xiang Yang Wang[3], Bin Chen[1,2*]

[1] Chongqing Key Laboratory of Non-linear Circuit and Intelligent Information Processing, College of Electronic and Information Engineering, Southwest University, Chongqing 400715, China, hanhanblala@email.swu.edu.cn

[2] Key Laboratory of Luminescent and Real-Time Analytical Chemistry (Southwest University) Ministry of Education, College of Pharmaceutical Sciences, Southwest University, Chongqing 400715, China.

[3] CET-College of Engineering and Technology, Southwest University, Chongqing 400715, China.

* Correspondence: Chenbin121@swu.edu.cn; Tel.: +86- 182-2310-5797

Table S1. Typical electrochemical-based sensors for detection of disease-related biomolecules

Analyte	Principle	Advantages/Limitations	LOD	Reference
Antibodies to tTG	Gold-based self-assembled monolayers based on biopodal alkane thiols for immobilization of tTG	low concentration of human IgA antibodies	390 ng/mL	[70]
Antibodies to tTG	Guinea pig protein as the antigen to detect antibodies of tTG in human serum	Rapid binary response, robust, user-friendly, cost-effective/ higher number of clinical samples are needed to be process	-	[72]
Anti-hepatitis B virus antibodies	A novel magnetosandwich assay-based biosensor based on AuNPs labels	Simple, low cost, allow its application at doctor-office, lower sample volumes required	3 mIU/mL	[6]
Hepatitis B virus surface antigen	Fe_3O_4 magnetic nanoparticles as carriers, Au sheet as working electrode, heminG0quadruplex DNAzyme and gold NPs as signal amplifier	Ultrasensitive, simplicity, reliability	0.19 pg mL^{-1}	[66]
CEA	Ag/Au nanoparticles to coat on graphene as signal amplifying factor	High sensitivity and selectivity, wide linear response range / the time of analysis is a critical factor	8 pg mL^{-1}	[82]
CEA	Nanogold encapsulated HRP label conjugated to the secondary anti-CEA antibody in a sandwich enzyme immunoassay format	Sensitive, only a little instrumentation are required	1.5 ng mL^{-1}	[83]

Table S1. *Cont.*

CEA and AFP	Uses metal ions tagged immunocolloidal gold nanocomposites as signal tags	Good sensitivity and selectivity	4.6 pg mL^{-1} for CEA, 3.1 pg mL^{-1} for AFP	[85]
CEA	Uses three-dimensional graphene foam as electrode architecture for immunosensing of tumor biomarkers	Accessible to bioaffinity ligands, high density of the immobilized antibody, high electrode conductivity, robust, fast, and sensitive	90 pg ml^{-1}	[86]
CEA	Employs three kind of lectins as molecular recognition elements, and gold nanoparticles as platform, horseradish peroxidase functionalized antibody as signal probe, for dual signal amplification	Low detection limit, good sensitivity, wide linear range	0.03 ng mL^{-1}	[87]
Prostate specific antigen (PSA)	Uses/immunochromatographic electrochemical biosensor that is based on nanoparticle label	Rapid, sensitive, reproducible, clinically practical	0.02 ng mL^{-1}	[92]
B. anthracis Sap antigen	Uses Au-Pd NPs@BNNs nanohybrid for redox cycle based detection	High catalytic activity, fast	1 pg mL^{-1}	[93]
PSA	Uses an immunosensor based on three different generations of ferrocene cored polyamidiamine dendrimers gold electrode	Sensitive, selective and disposable, excellent performance for PSA at the pulse amplitude	0.001 ng mL^{-1}	[91]
PSA	Uses gold nanospears that has been electrodeposited as a transducer to immobilize an aptamer of PSA	High surface area to immobilize a high surface concentration of the aptamer sequence, high sensitivity, applicable for clinical analysis	50 pg mL^{-1}	[94]
Folate receptor (FR)	Adopts homogeneous indium tin oxide (ITO)-based electrochemical detection	Simple, convenient immobilization-free ITO-based detection strategy, high selectivity of the terminal protection of small molecule linked DNA	3.8 fM	[96]

Table S1. *Cont.*

DNA sequences related to herpes simplex viruses (HSV)	Uses Meldola Blue as the hybridization indicator for detection and discrimination of HSV Type Ⅰ and Ⅱ	Fast, reliable, and low-cost detection	14.78 fM for probe 1 and 14.77 fM for probe 2	[33]
Influenza B virus	Uses AuNPs-modified disposable sensor, and Meldola's blue was used as intercalator label for detection of influenza B virus	Rapid, simple, and sensitive	3.3×10^{7} molecules in 37 min	[89]
SNPs	Uses disposable electrochemical printed chips connected with redox active molecule Hoechst 33258 to detect SNPs from unpurified PCR amplicons	Inexpensive, rapid and hand-held detection systems	-	[106]
dsDNA corresponding to hepatitis C virus genotype 3a	Uses gold electrode modified with a SAM composed of the PNA probe and MCH to detect ds-DNA corresponding to HCV3a via triplex formation	Provides the possibility of monitoring the hybridization of the PNA probe with the target dsDNA	1.8×10^{-12} M	[108]
Breast cancer related ssDNA	Use $[Fe_3(CN)_6]^{-3/-4}$ as an electrochemical redox couple	Excellent selectivity for discriminating complementary sequences	4.6×10^{-20} M	[3]
Influenza virus (type A) related DNA sequence	Uses an E-DNA biosensor by chemical reduction and avidin-biotin conjugation on the surface of a GC electrode for detection of a DNA sequence-specific target DNA	High sensitivity, wide dynamic detection range, effectively discriminates between a completely complementary target sequences	8.51×10^{-14} M	[109]
Salmonella	Thiolated *Salmonella* aptamer ssDNA sequence linked to a GCE electrode, which has been modified with GO and electrode-posited with GNPs	High specificity and selectivity to aptamer, fast	3 cfu/mL	[112]
Targeted DNA species related to oral cancer	Uses nicking endonuclease assisted target recycling and the differential diffusivity between electroactive reporter-tagged long DNA and short DNA toward a negatively charged ITO electrode surface for signal amplification.	Simple, rapid, immobilization-free, ultrahigh selective	0.35 pM	[103]

Table S1. *Cont.*

microRNA	Based on the mismatched catalytic hairpin assembly (CHA) amplification to decrease the background reaction signal	High specificity, low background signal	0.6 pM	[115]
microRNA	Uses a label-free miRNA biosensor based on a novel isothermal signal amplification strategy	The simplicity of the one-step label-free detection, isothermal amplification process	1.0 fM	[116]
Oral cancer-related miRNA	Based on an electrically magnetic-controllable gold electrode with "junction-probe" strategy and MBs-based enzymatic catalysis amplification	The strength and direction of the magnetic field and the temperature of the electrode's surface can be easily regulated	2.2×10^{-19} M	[113]
miRNA-21	Involves a sandwich hybridization assay onto gold nanoparticles modified GCE and enzyme signal amplification	Promising sensing platform, allows the good capture of miRNA-21 target, high sensitivity	100 pM	[118]
Fructosyl valine (FV)	Uses a single-use, disposable biosensor, which prototype was fabricated using thick film screen-printing technique, for the detection of FV	Single-use, disposable, high reproducibility	1 µM	[126]
Thrombin and adenosine	Uses a switching structures of aptamers from DNA/DNA duplex to DNA/target complex using MB as an indicator	Simple, convenient, cost effective	3 nM for thrombin and 10 nM for adenosine	[128]
MUC1	A hairpin oligonucleotide (HO) switch, gold nanoparticles, and enzyme signal amplification	High specificity, low LOD, and wide linear range	2.2 nM	[130]
CEA and AFP	Uses a sandwich-format immunosensor using metal ions tagged immunocolloidal gold nanocomposites as signal tags	Good stability, wide linear working ranges were in good agreement with standard ELISA	4.6 pg mL^{-1} (CEA) and 3.1 pg mL^{-1} (AFP)	[85]
Vascular endothelial growth factor	Uses a folding-based electrochemical aptasensor for detection of vascular endothelial growth factor	Cost-effective, single-use, sensitive, complex media selectivity, and reusability	5pM (190pg/mL)	[138]

Table S1. *Cont.*

Adenosine and lysozyme	Uses a bifunctional biosensor for detection of adenosine or lysozyme by virtue of switching structures of aptamers from DNA/DNA duplex to DNA/target complex	Good selectivity, reproducibility, and stability	0.01 μg mL^{-1} for lysozyme, 0.02 nM for adenosine	[142]
